# Integrating single‐cell and multi‐omic approaches reveals Euphorbiae Humifusae Herba‐dependent mitochondrial dysfunction in non‐small‐cell lung cancer

**DOI:** 10.1111/jcmm.18317

**Published:** 2024-05-27

**Authors:** Chengcheng Zhang, Xiaoxue Zhao, Feng Li, Jingru Qin, Lu Yang, Qianqian Yin, Yiyi Liu, Zhiyao Zhu, Fei Zhang, Zhongqi Wang, Haibin Liang

**Affiliations:** ^1^ Department of Medical Oncology Longhua Hospital affiliated to Shanghai University of Traditional Chinese Medicine Shanghai China; ^2^ Department of Rheumatology Longhua Hospital affiliated to Shanghai University of Traditional Chinese Medicine Shanghai China; ^3^ Department of General Surgery Xinhua Hospital Affiliated to Shanghai Jiao Tong University School of Medicine Shanghai China

**Keywords:** EHH, immune, mitochondrial dysfunction, multi‐omics, NSCLC, single‐cell

## Abstract

Euphorbiae Humifusae Herba (EHH) is a pivotal therapeutic agent with diverse pharmacological effects. However, a substantial gap exists in understanding its pharmacological properties and anti‐tumour mechanisms. This study aimed to address this gap by exploring EHH's pharmacological properties, identifying NSCLC therapy‐associated protein targets, and elucidating how EHH induces mitochondrial disruption in NSCLC cells, offering insights into novel NSCLC treatment strategies. String database was utilized to explore protein–protein interactions. Subsequently, single‐cell analysis and multi‐omics further unveiled the impact of EHH‐targeted genes on the immune microenvironment of NSCLC, as well as their influence on immunotherapeutic responses. Finally, both in vivo and in vitro experiments elucidated the anti‐tumour mechanisms of EHH, specifically through the assessment of mitochondrial ROS levels and alterations in mitochondrial membrane potential. EHH exerts its influence through engagement with a cluster of 10 genes, including the apoptotic gene CASP3. This regulatory impact on the immune milieu within NSCLC holds promise as an indicator for predicting responses to immunotherapy. Besides, EHH demonstrated the capability to induce mitochondrial ROS generation and perturbations in mitochondrial membrane potential in NSCLC cells, ultimately leading to mitochondrial dysfunction and consequent apoptosis of tumour cells. EHH induces mitochondrial disruption in NSCLC cells, leading to cell apoptosis to inhibit the progress of NSCLC.

## 1 INTRODUCTION

The effects of cancer are influenced by a myriad of factors, including epigenetic mechanisms,^1^ microbial populations^2^ and the surrounding immune cellular environment.^3^ These elements bestow upon neoplastic cells the abilities of resistance to pharmacological treatments and enhanced proliferative migration, often leading to adverse prognoses for patients.^4^, ^5^ Traditional Chinese Medicine (TCM) has long been utilized in addressing a variety of health issues, thereby providing a rich source of bioactive entities for potential utilization in pharmacological innovation. An exemplar of this is the Euphorbiae Humifusae Herba (EHH), a prominent TCM formulation widely adopted in Eastern Asia for a prolonged period, known as Di‐Jin‐Cao in China and Ttang‐Bin‐Dae in Korea.^6^

EHH exhibits a multitude of pharmacological properties, encompassing anti‐tumour and antioxidant,^6^, ^7^, ^8^, ^9^, ^10^ and anti HBV.^11^, ^12^ In recent years, extensive research has delved into the role of EHH in cancer therapy, with numerous investigations confirming its anti‐cancer activity. EHH prompts apoptosis in tumour cells, a programmed cellular death process. Facilitating apoptosis contributes to halting uncontrolled proliferation of cancer cells. Furthermore, EHH can inhibit the division and proliferation of cancer cells, consequently decelerating tumour growth. Tumour growth and dissemination are typically accompanied by angiogenesis to supply the required nutrients and oxygen. EHH exhibits anti‐angiogenic properties, preventing neovascularization and thereby constraining tumour progression.^13^ Through immune system modulation, EHH assists in diminishing the tumour cells’ capacity to evade immune surveillance. These anti‐tumour effects of EHH are contingent upon the disruption of several intracellular signalling pathways within cancer cells, including PI3K/Akt, MAPK, NF‐κB and more. EHH's immune modulation encompasses augmenting immune cell activity and promoting cytokine production, contributing to an enhanced immune response against cancer within the body. Although the anti‐tumour potency of EHH is well‐recognized, the detailed understanding of its mechanistic pathways is not yet fully deciphered. Significant research focus has been placed on exploring the therapeutic possibilities of EHH in various cancers, including lung and breast cancers.^6^, ^13^, ^14^ EHH, renowned for its anti‐inflammatory properties and prospective anti‐tumoral impact, has garnered considerable attention. Nonetheless, a comprehensive understanding of the intricate molecular pathways that contribute to EHH's influence, and the consequent modifications in cellular phenotype, is an area necessitating further investigative scrutiny.

Mitochondrial dysfunction refers to abnormalities in the biochemical reactions and energy production processes within mitochondria. Typically, it manifests as aberrations in biological functions such as reduced ATP synthesis and disturbances in redox balance. This type of functional impairment is widely recognized to be closely associated with the development and progression of various tumours, as mitochondria play pivotal roles in regulating essential biological processes including cell growth, apoptosis and oxidative stress.^15^ Furthermore, mitochondrial dysfunction can also impact the normal functioning of the immune system, resulting in decreased immune cell activity, increased cell death and inappropriate activation of inflammatory responses, thereby creating a more favourable microenvironment for tumour development. In‐depth exploration of the interplay between mitochondrial dysfunction and tumours, as well as its implications for immune responses, holds significant importance in advancing our understanding of tumour biology and the development of relevant therapeutic strategies.

In this study, we embarked on a systematic exploration of EHH's intrinsic biological properties. Simultaneously, we leveraged the resources provided by the BindingDB repository to identify potential protein targets associated with EHH. Our investigation of the Genecards database yielded a compilation of 5815 prospective therapeutic targets relevant to NSCLC. Further, utilizing the application of single‐cell analysis and multi‐omic analysis, we elucidated the anti‐tumour target points of EHH. Meanwhile, in vitro experiments confirmed that EHH induces mitochondrial disruption in NSCLC cells, leading to apoptosis. This sheds new light on the anti‐tumour mechanisms of EHH and underscores the pivotal role of targeting mitochondrial dysfunction in NSCLC therapy.

## 2 MATERIALS AND METHODS

### 2.1 Evaluation of pharmacokinetic properties using TCMSP

The TCMSP platform, accessible via http://lsp.nwu.edu.cn/tcmsp.php, serves as an extensive database primarily centred on medicinal components and their chemical elements.^16^, ^17^ This resource operates in a structured manner, offering detailed perspectives on the complex dimensions of absorption, distribution, metabolism and excretion (ADME) characteristics inherent to bioactive agents. These characteristics include essential parameters like oral bioavailability (OB), drug‐likeness (DL), Caco‐2 permeability and blood–brain barrier penetration, crucial for understanding the pharmacokinetic and pharmacodynamic actions of these substances in biological contexts.^18^, ^19^ Our research concentrates on ‘Euphorbiae Humifusae Herba’, keyed into the search field, aiming for a thorough examination of its pharmacokinetic attributes at a molecular level. The ensuing analysis focuses on selecting compounds with OB values of 30% or more and DL values of at least 0.18, setting the stage for an extensive evaluation.

### 2.2 Identification of EHH targets

Files in mol2 format, corresponding to a group of 13 compounds screened for EHH, were obtained with precision through extraction methods from the TCMSP database. After a thorough review of relevant literature, elements showing no significant target interactions in the TCMSP database were selectively removed. This methodical selection led to the recognition and confirmation of seven unique active substances inherent to EHH, enhancing the academic integrity and systematic consistency of the research methodology.

### 2.3 Genecards for identifying disease target

In this study, the utilization of Genecards (https://www.genecards.org/) proves integral, given its role as a repository replete with disease‐associated targets.^20^, ^21^ Our investigative pathway involves harnessing the Genecards online tool to discern potential therapeutic targets within the framework of NSCLC. By initiating a query employing the keyword ‘NSCLC’ and subsequently filtering the results through the ‘evidence of genetic disease association’ criterion, a corpus of 23,953 relevant targets is amassed. The winnowing process hinges upon the ‘correlation score’, imposing a condition wherein a correlation score exceeding twofold holds significance. Through the judicious elimination of duplicate genetic entities, a curated cohort of 5815 targets emerges, poised for subsequent meticulous explorations.

### 2.4 Advancement in establishing a collective network model for EHH‐NSCLC

The open‐source tool, Cytoscape 3.7.2, found at the URL http://www.cytoscape.org/, is specifically engineered for the graphical representation of complex bio‐molecular networks and integrating various types of attribute data.^22^, ^23^ Leveraging Cytoscape 3.7.2, we construct a shared target network for the visual representation of EHH‐NSCLC, drawing from the aforementioned dataset. This network adeptly encapsulates the intricate interplay existing between EHH and NSCLC, along with their interrelated targets. Within this network, distinct nodes depict EHH, NSCLC and the corresponding linked targets. The interconnections between these nodes distinctly portray the interactions underpinning these biological analyses. Notably, the molecular degree value directly corresponds to the count of connections established amidst molecules and targets within this network.

### 2.5 Investigating core gene expression in immune cells via single‐cell sequencing techniques

As an integrated database and access point, the TISCH2 Portal facilitates the exploration of individual cell transcriptomes related to the tumour immune microenvironment.^24^ Through the utilization of this portal, a comprehensive evaluation of 10 critical genes' expression profiles within immune cells was performed. Considering the scenario of distal metastasis in NSCLC, a unique approach to single‐cell sequencing was employed. This approach included a study of NSCLC patients exhibiting signs of distal metastasis.^25^, ^26^

### 2.6 Evaluation of immunotherapy efficacy

Considering the intricate linkages among a core group of 10 genes in immune cells, a question emerges about their effect on the reactivity of patients with non‐small‐cell lung cancer (NSCLC) to immunotherapy treatments. This study explores the role of these 10 crucial genes in the modulation of immune checkpoint markers, while simultaneously shedding light on their involvement in immunological pathways by applying gene ontology (GO) and Kyoto Encyclopedia of Genes and Genomes (KEGG) analytical methods.

### 2.7 Core target molecular dock

In the study, molecular docking techniques were applied to verify the affinity of binding between EHH and the crystal structure of the protein targeting the nucleus. The three‐dimensional structure of the EHH's nucleus‐targeting site, relevant to the treatment of NSCLC, was determined by accessing data from the RCSB PDB database (https://www.rcsb.org/).^27^ Correspondingly, the tridimensional structures of molecular ligands were sourced from the PubChem database (https://pubchem.ncbi.nlm).^28^ Subsequent to the retrieval, the protein–ligand complex was processed through Autodock4.2, encompassing procedures for the elimination of aqueous molecules, hydrogenation and computation of the protein's net charge. The most optimal conformation ensuing from the docking process was selected on the basis of its potent binding affinity, subsequently visualized using PyMOL 2.4.2. The dehydration of the receptor protein was executed via the PyMOL 2.4.2 software, while protein hydrogenation and charge calculations were carried out using Autodock software. Configurations of the receptor protein's docking sites were fine‐tuned to encompass the active pocket region, specifically designed to accommodate interactions with small molecule ligands. Ultimately, the docking operation between the receptor protein and the small molecular ligand of the EHH active substance was facilitated by Autodock Vina. As a general criterion, binding energies of ≤−5 kJ/mol were adopted as the threshold for selection. Noteworthy PDB IDs encompassed 1flt, 3poz and 6G6L, respectively. In instances where a suitable ligand for the pertinent protein was unattainable, the online platform playmolecule (https://www.playmolecule.com/deepsite/) was enlisted to forecast docking locales.^29^ The prognosis obtained through this predictive method was subsequently regarded as the central point for the docking pocket.

### 2.8 Cytotoxicity of EHH

We obtained the murine NSCLC cellular model, specifically the Lewis lung carcinoma (LLC) line, via the Chinese Academy of Sciences' Cell and Stem Cell Repository. These cells were cultured in a Dulbecco's Modified Eagle Medium (DMEM, catalogue #L110KJ, Basalmedia Technologies) and enriched with a 10% solution of foetal bovine serum (FBS, catalogue #10099141C, Basalmedia Technologies). The maintenance of cell cultures was conducted at a constant temperature of 37°C in an atmosphere containing 5% CO2, using a specialized CO2 incubator. To initiate the subcutaneous xenograft paradigm, we injected LLC cells (quantity: 2 × 10^5^ cells per mouse) into the inguinal area of 6‐week‐old female C57BL/6J mice, sourced from Shanghai University of Traditional Chinese Medicine. We regularly measured the tumours' dimensions, including length and width, at 3‐day intervals, calculating the tumour volume accordingly. Further, we employed immunohistochemical (IHC) staining and HE staining as methodologies for the toxicological examination of EHH's effects. Immunohistochemical staining was performed according to the following protocol: Initially, tissue sections that had been formaldehyde‐fixed and paraffin‐embedded were subjected to sequential treatments. The sections underwent deparaffinization with xylene and were rehydrated through a gradient alcohol series, followed by antigen retrieval. Subsequently, a 60‐min blocking step was carried out with 5% bovine serum albumin (BSA).^30^ Following this, the sections were incubated overnight at 4°C with primary antibodies. The primary antibodies employed encompassed anti‐Vimentin (diluted 1:200) and anti‐E‐cadherin (diluted 1:200) antibodies.^31^

### 2.9 Measurement of mitochondrial dysfunction

NCI‐H1299 and A549 cell lines were obtained from Chinese Academy of Sciences' Cell and Stem Cell Repository. In our investigation, we utilized MitoSOX™ Red (Invitrogen, USA) as a mitochondrial superoxide indicator to assess the production of mitochondrial ROS within NCI‐H1299 cells. After a 10‐min incubation period with 50 μM MitoSOX Red, we proceeded to the next step. Subsequently, the cells were subjected to a 30‐min incubation in darkness with JC‐1 (2 μg/mL) to determine the mitochondrial membrane potential (ΔΨm). This evaluation was carried out using the JC‐1 MitoMP Detection Kit (Dojindo, Japan), following the manufacturer's instructions.

### 2.10 CCK‐8 assay

Different EHH concentrations were applied to the cell cultures of both A549 and NCI‐H1299. Following a 48‐h period of incubation, each well received 10 μL of CCK‐8 reagent (sourced from Jindo, Japan) and was further incubated for 2 h.^32^, ^33^

### 2.11 Western blot

Post‐exposure of NCI‐H1299 cells to EHH, proteins were isolated employing a lysis buffer specific for proteins. The procedure for western blotting, a commonly used technique in molecular biology, was then meticulously followed, culminating in the transfer of the extracted proteins to a polyvinylidene difluoride (PVDF) membrane. This was succeeded by a 60‐min treatment using a buffered solution of bovine serum albumin (BSA). Primary antibodies, specific to Caspase 3 and Actin, were then introduced. This was followed by the addition of a diluted secondary antibody, and the incubation was extended for an additional hour. The detection of proteins was carried out subsequently. Analytical quantification of the protein expression levels was conducted utilizing the ImageJ software.

### 2.12 Statistical analysis

In this investigation, GraphPad Prism 8 software was employed for all statistical analyses. Data presentation encompasses mean values along with their corresponding standard error of the mean (SEM). To evaluate differences among pairs or various groups, we applied either the Student's *t*‐test or the analysis of variance (ANOVA) method. Significance in each case was established at *p* < 0.05, denoting statistical relevance.

## 3 RESULTS

### 3.1 Screen the bioactive compounds

**FIGURE 1:**
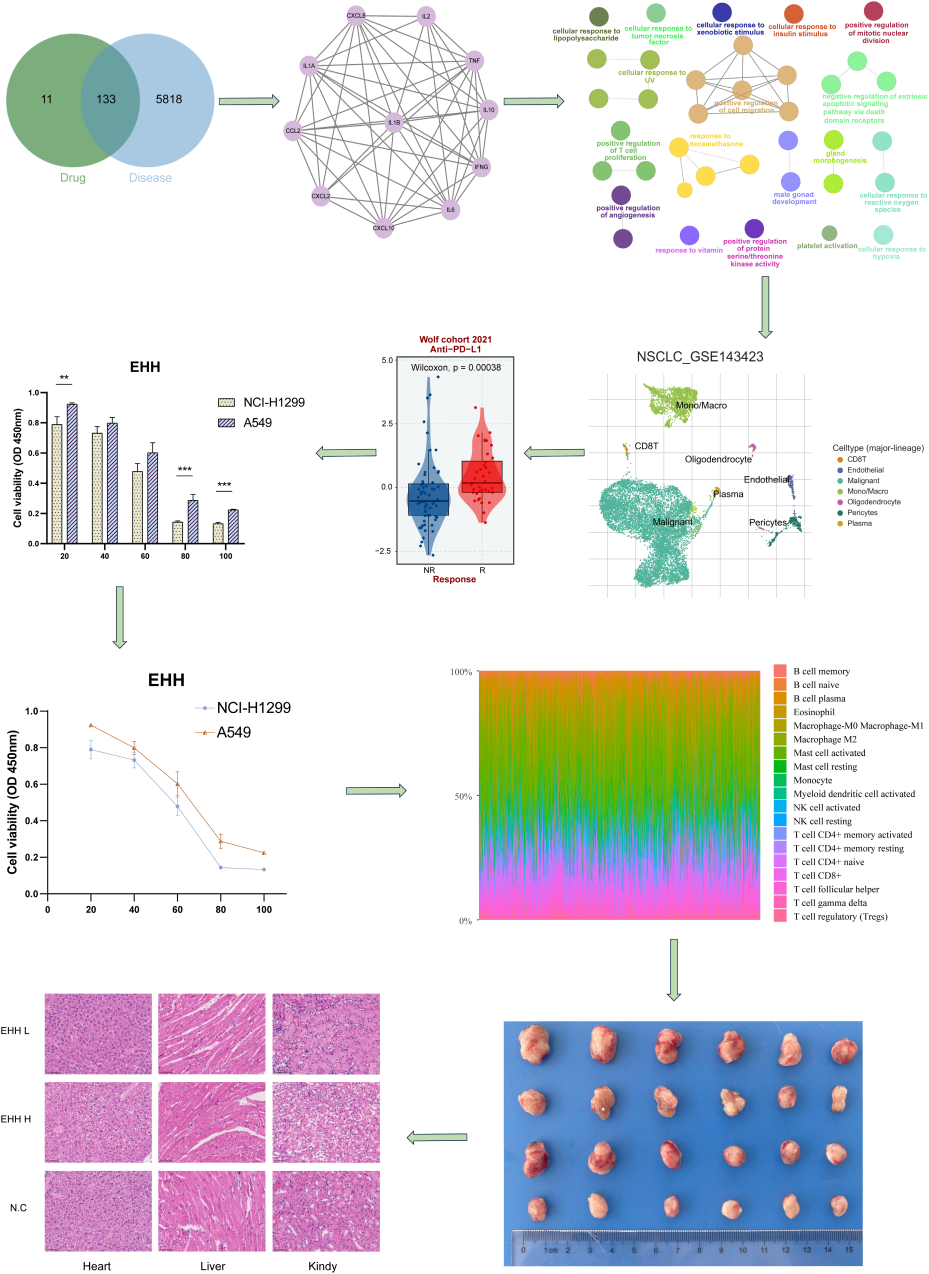
Schematic diagram. (**p < 0.01, ***p < 0.001).

In the current study, a meticulous methodology was utilized for the identification of bioactive agents (Figure 1). The Traditional Chinese Medicine Systems Pharmacology (TCMSP) database was the source for Euphobrase Humifusae Herba, from which 38 unique compounds were isolated. In alignment with the scholarly standards set by journals such as Nature and Cell, the evaluation process for these compounds centred on their pharmacokinetic attributes, focusing primarily on oral bioavailability (OB) and drug‐likeness (DL). The criteria for selection were stringent, necessitating a baseline OB of no less than 30% and a DL index surpassing the threshold of 0.18. Utilizing these parameters, a streamlined group of 13 chemical entities was identified. Emphasizing the complexities inherent in pinpointing targets is essential. Remarkably, within the Traditional Chinese Medicine Systems Pharmacology (TCMSP) database, six of these entities lacked identifiable targets, indicating a gap in target‐related data. Therefore, through careful selection, a group of seven chemical entities, representing about 10% of the initial group, was curated. Each of these seven entities, linked to relevant targets, underwent a thorough investigative process. In Table S1, a detailed presentation of chosen chemical entities, their characteristics and relevant target data is provided. The ensuing stages of analysis, centred on these entities, possess the capability to generate significant revelations concerning their medicinal uses and operational modalities.

### 3.2 Elucidation of EHH's therapeutic targets in NSCLC

**FIGURE 2:**
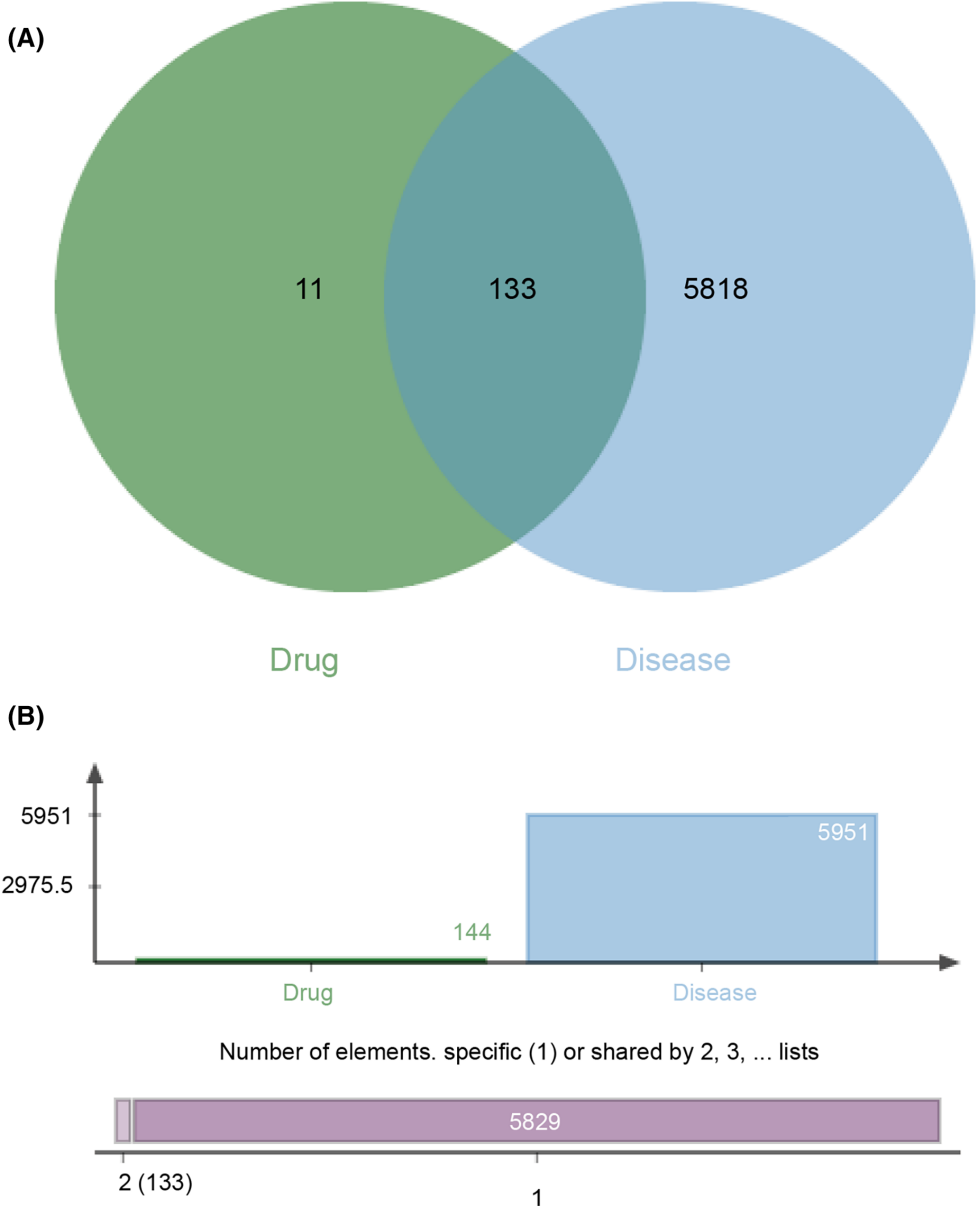
Screening for common genes. (A) The common 133 target genes for EHH and NSCLC. (B) Number of genes, regarding EHH target genes, and differential genes in NSCLC.

Through the application of the TCMSP computational framework, as detailed in our methodology, we embarked on the elucidation of potential molecular targets associated with EHH's seven efficacious agents. This process involved interrogating the NCBI's Gene Database to acquire the relevant official gene symbols and IDs. Following a thorough exercise in deduplication, we identified a unique assembly of 144 target genes. These genes, influenced by the seven potent compounds of EHH, laid the groundwork for future investigative paths (Figure 2A). In an extensive assemblage process, 23,953 interlinked target entities were aggregated following a methodical investigation using ‘non‐small‐cell lung cancer’ as the query in the Genecards database (genecards.org). The calibration of screening thresholds was meticulously adjusted, employing the ‘correlation score’ as a pivotal criterion. This elaborate curation process, which included the elimination of superfluous gene entries, resulted in a distilled collection of 5951 target entities, as depicted in Figure 2B. Such a stringent refinement procedure lays a solid groundwork for further investigative stages.

### 3.3 Protein–protein interaction

**FIGURE 3:**
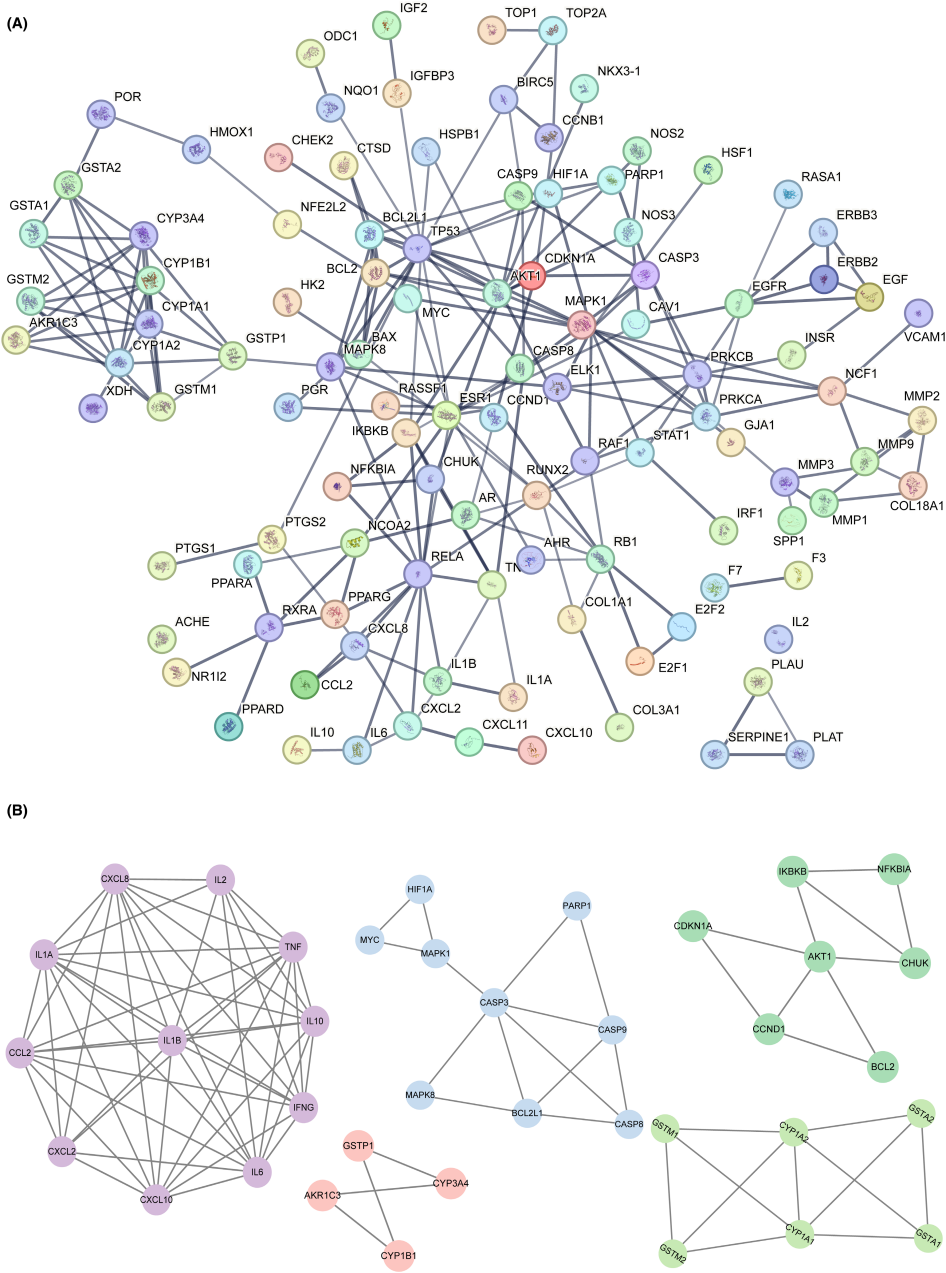
Interaction networks of EHH‐targeted genes and core genes (A) Protein–protein interactions exhibits the networks of EHH‐targeted genes. (B) 5 Core networks of PPI.

**FIGURE 4:**
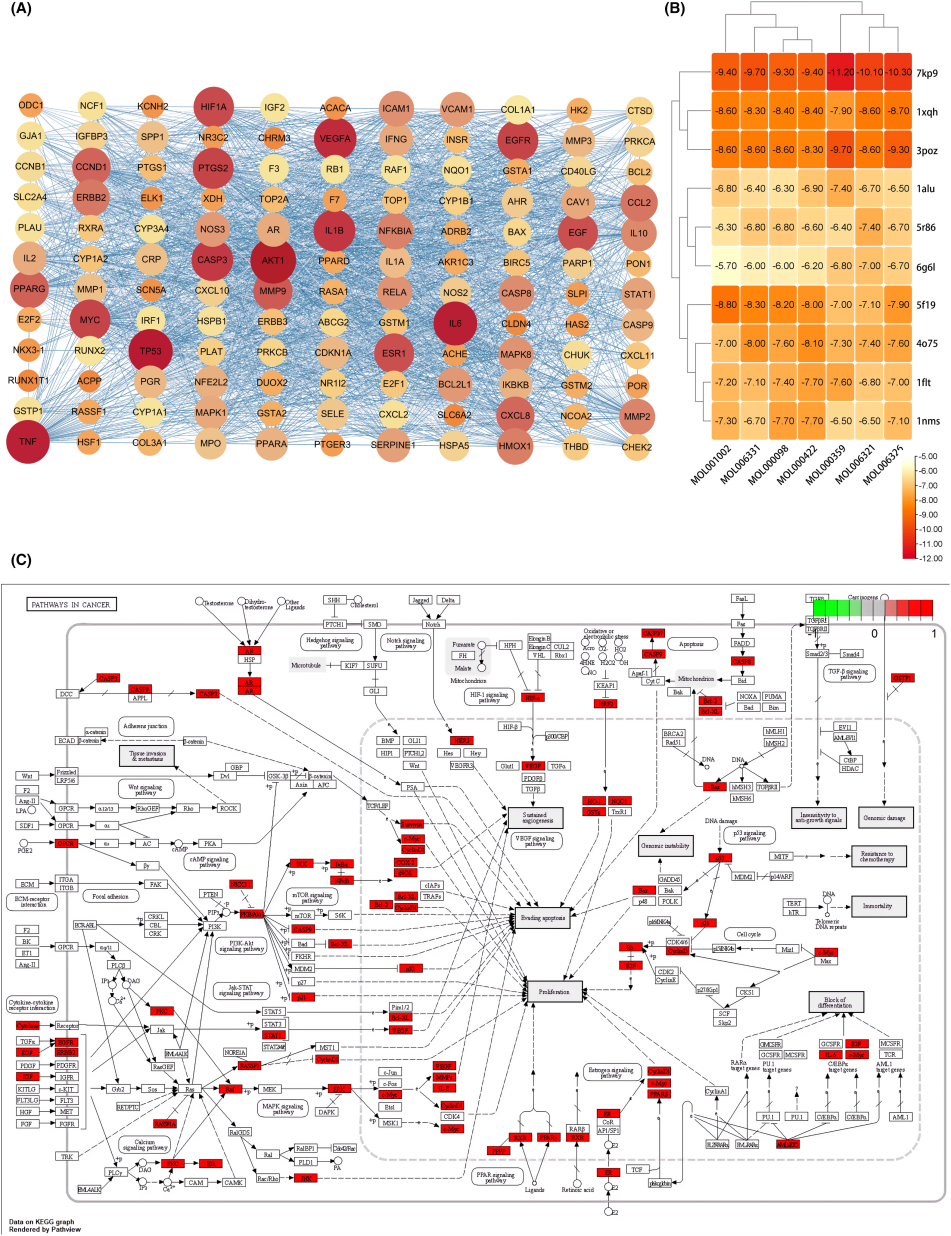
KEGG and signalling pathways. (A) Regulatory networks of EHH‐targeted genes. (B) Seven active components and 10 core target proteins of EHH. (C) Core signalling pathway induced by EHH.

We successfully pinpointed 133 genes that are common between the datasets of EHH and NSCLC (Figure 3). Utilizing Cytoscape software version 3.7.2, a comprehensive visual representation of the combined EHH‐NSCLC gene network was formulated, grounded on the datasets mentioned earlier. Moreover, we developed a network specifically for protein–protein interactions (PPI) integrating these overlapping genes, which incorporates exactly 133 nodes (Figures 3A and 4A). Remarkably, this network of protein interactions is characterized by 133 interconnected nodes and a total of 2309 linking edges. The nodes exhibit an average degree of 34.7, along with an average local clustering coefficient standing at 0.69. The significance of PPI enrichment is markedly low, with a *p*‐value significantly under 1.0e‐16, detailed in Figure S1. In addition, our analysis revealed a cluster of five key networks (Figure 3B).

We scrutinized the PPI network, emphasizing genes pivotal in communication. Our rigorous approach led to the identification of 10 paramount genes. These genes were chosen adhering to stringent guidelines: Both the BC and CC median values had to surpass the threshold of 2. Our comprehensive analysis yielded a list of critical genes, namely AKT1, IL6, TP53, TNF, IL1B, EGFR, MYC, CASP3, VEGFA and PTGS2, as detailed in Table S2. To investigate the molecular interactions among the seven principal constituents of G. lucidum and their affinity towards 10 pivotal target proteins, molecular docking methodologies were utilized (Figure 4B). Remarkably, the pathway identified as most significant through KEGG enrichment analysis corresponds to the Hsa05200 pathway, which is frequently linked with the advancement of cancer (Figure 4C).

### 3.4 Immune cell core gene expression patterns as unveiled by single‐cell analysis

**FIGURE 5:**
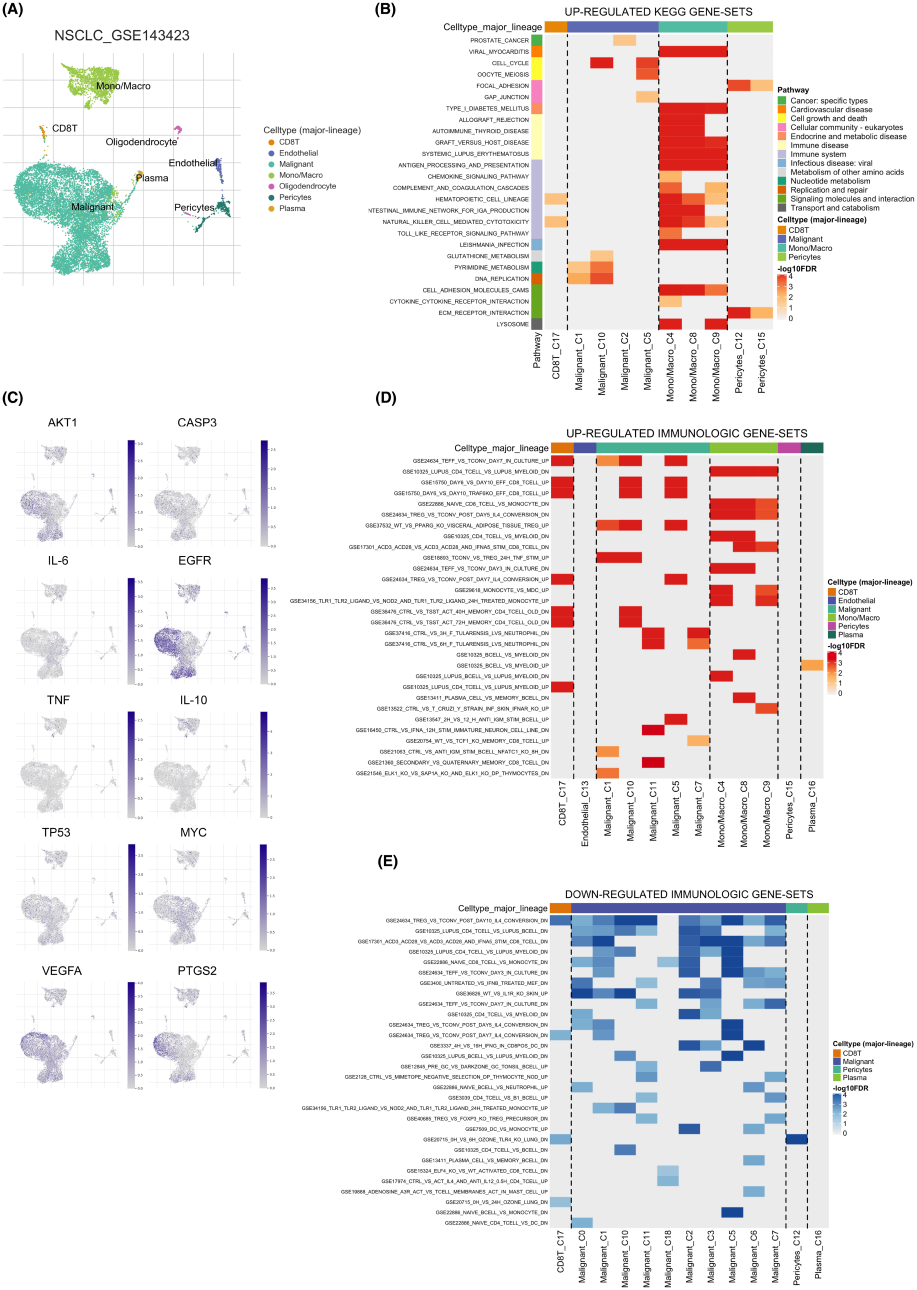
Single‐cell sequencing analysis in NSCLC. (A) UMAP diagram showing cell subpopulations. (B) Up‐regulated KEGG genesets. (C) 10‐core genes expression levels in the cell subtypes of NSCLC. (D) Up‐regulated immunologic genesets in NSCLC. (E) Down‐regulated immunologic genesets in NSCLC.

**FIGURE 6:**
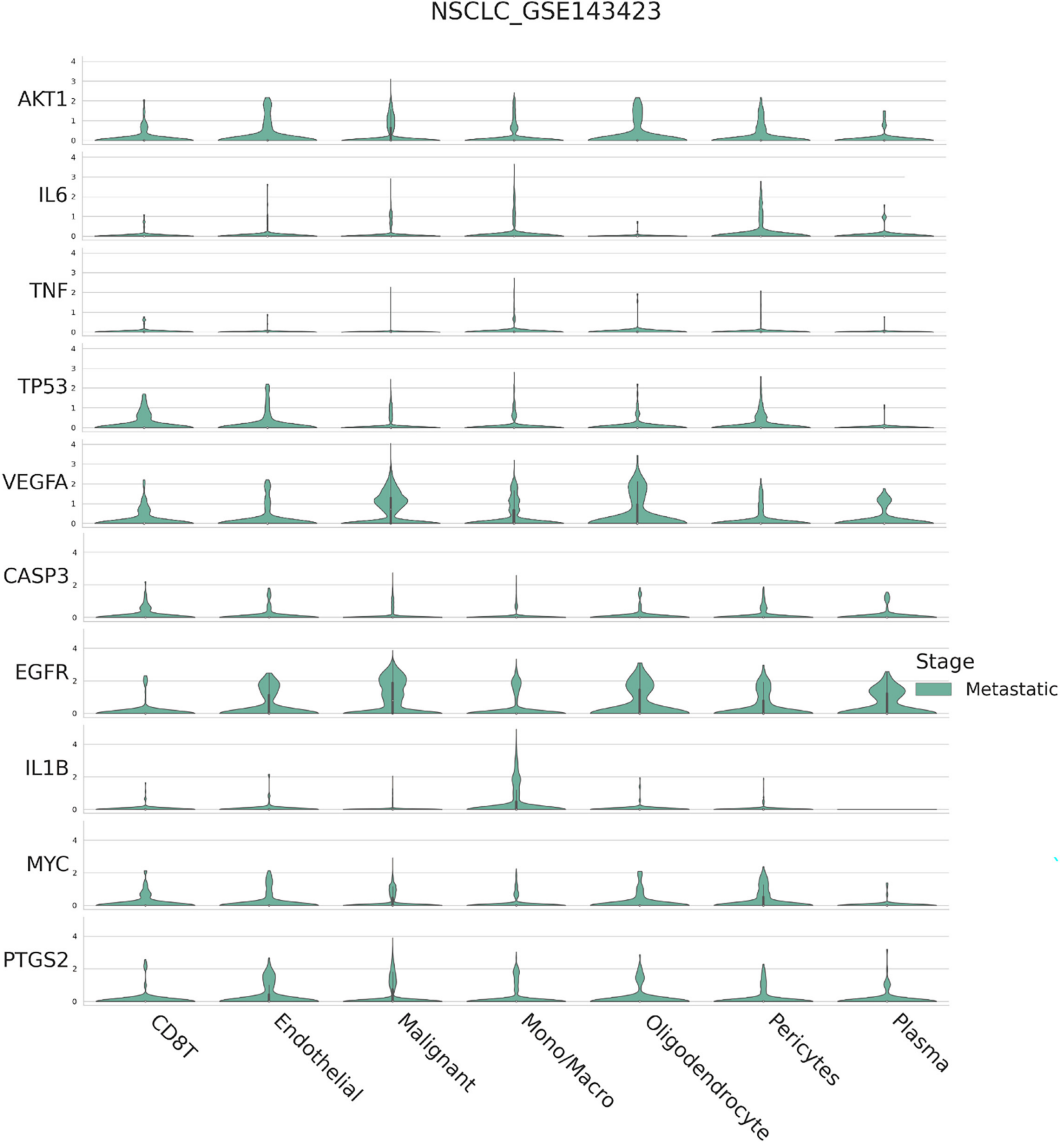
Single‐cell sequencing demonstrates expression patterns of 10 key genes in metastatic NSCLC.

In light of the profound influence exerted by the immune microenvironment on NSCLC development, our objective was to decode the expression signatures of 10 critical genes associated with EHH in immune cells. Our exploration uncovered that in NSCLC, CD8 T, Mono/Macro and Malignant cells represent the major segments (Figure 5A). Moreover, in the tumour microenvironment of NSCLC, the upregulation of CD8 T cells is closely associated with the pyrimidine metabolism and DNA replication pathways, while monocytes and macrophages are closely associated with the upregulation of lysosome and chemokine signalling pathway (Figure 5B). We further investigated the expression of 10 key genes in NSCLC cell subtypes, among which EGFR and VEGFA are significantly enriched in tumour cells (Figure 5C). Additionally, we explored the upregulation of immunologic genesets and the downregulation of immunologic genesets in the tumour microenvironment of NSCLC (Figure 5D,E). In advanced NSCLC, distant metastasis is a common complication. To explore this, we broadened our investigation, examining the expression of 10 pivotal genes in metastatic NSCLC cases. We observed pronounced elevation of VEGFA and EGFR levels in cancerous cells. Conversely, AKT1 was predominantly found in abundance in endothelial and oligodendrocyte cells. A particularly striking finding was the substantial presence of TP53 in CD8 T cells (Figure 6). Such findings suggest the potential role of EHH in influencing the progression of metastatic NSCLC through the regulation of these essential genes.

### 3.5 GO and KEGG pathway analysis

**FIGURE 7:**
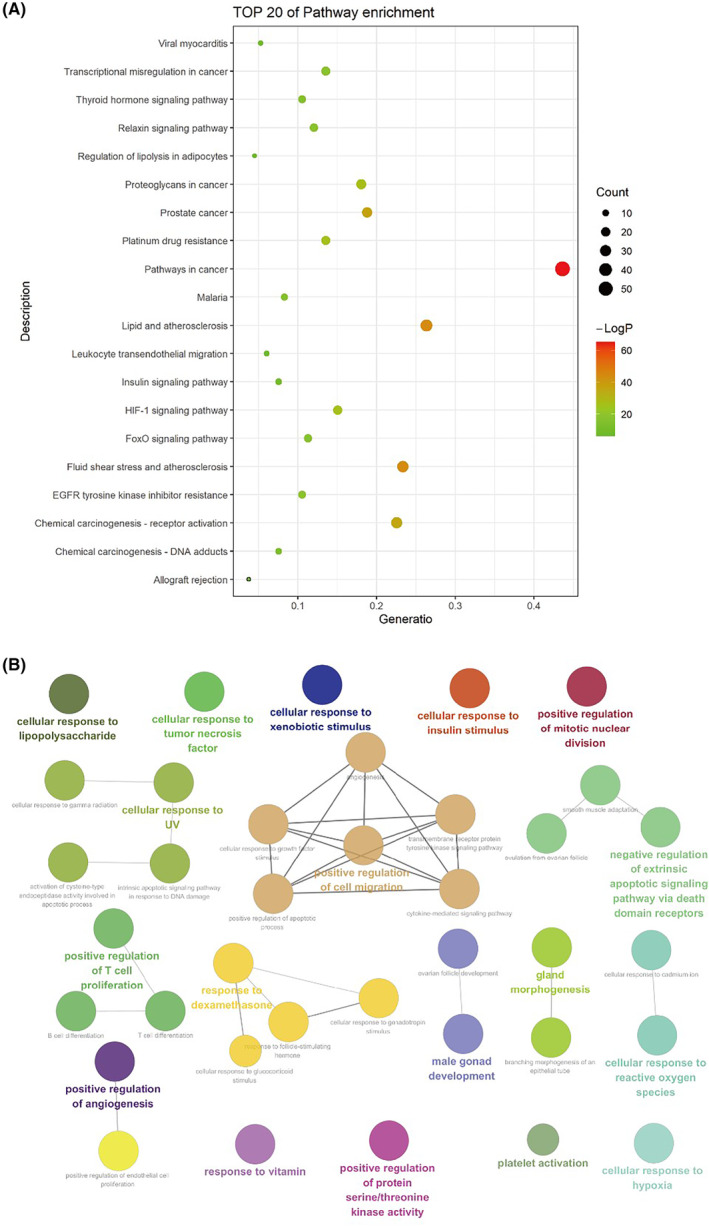
Gene Ontology (GO) and KEGG Pathway Analysis. (A) Top 20 of pathway enrichment involved by EHH‐targeted genes. (B) The association between pathways. (**p < 0.01, ***p < 0.001).

To deepen our understanding of the potential therapeutic roles of EHH in treating NSCLC, an extensive examination was carried out. Utilizing Metascape, this study conducted a bifurcated exploration, scrutinizing both Gene Ontology (GO) and KEGG pathway involvement. The focus of this research was on a specifically chosen group of 133 overlapping target genes, which formed the basis for meaningful conclusions. The results of this detailed study revealed a scenario predominantly characterized by pathways closely connected to cancerous activities, complex signalling within cells and the nuanced regulation of immune responses. A notable presentation of these findings is depicted in the graphical display seen in Figure 7A. Interestingly, the GO enrichment study highlighted a distinct pattern, revealing significant engagement of genes targeted by EHH in crucial cellular activities. In particular, noticeable tendencies were seen in the proliferation of T cells and the controlled movement of cells, aspects closely linked to the pathology of NSCLC. This significant finding (Figure 7B) aligns well with the 10 primary EHH‐related genes identified through an in‐depth analysis using single‐cell examination.

### 3.6 Immunotherapy response

**FIGURE 8:**
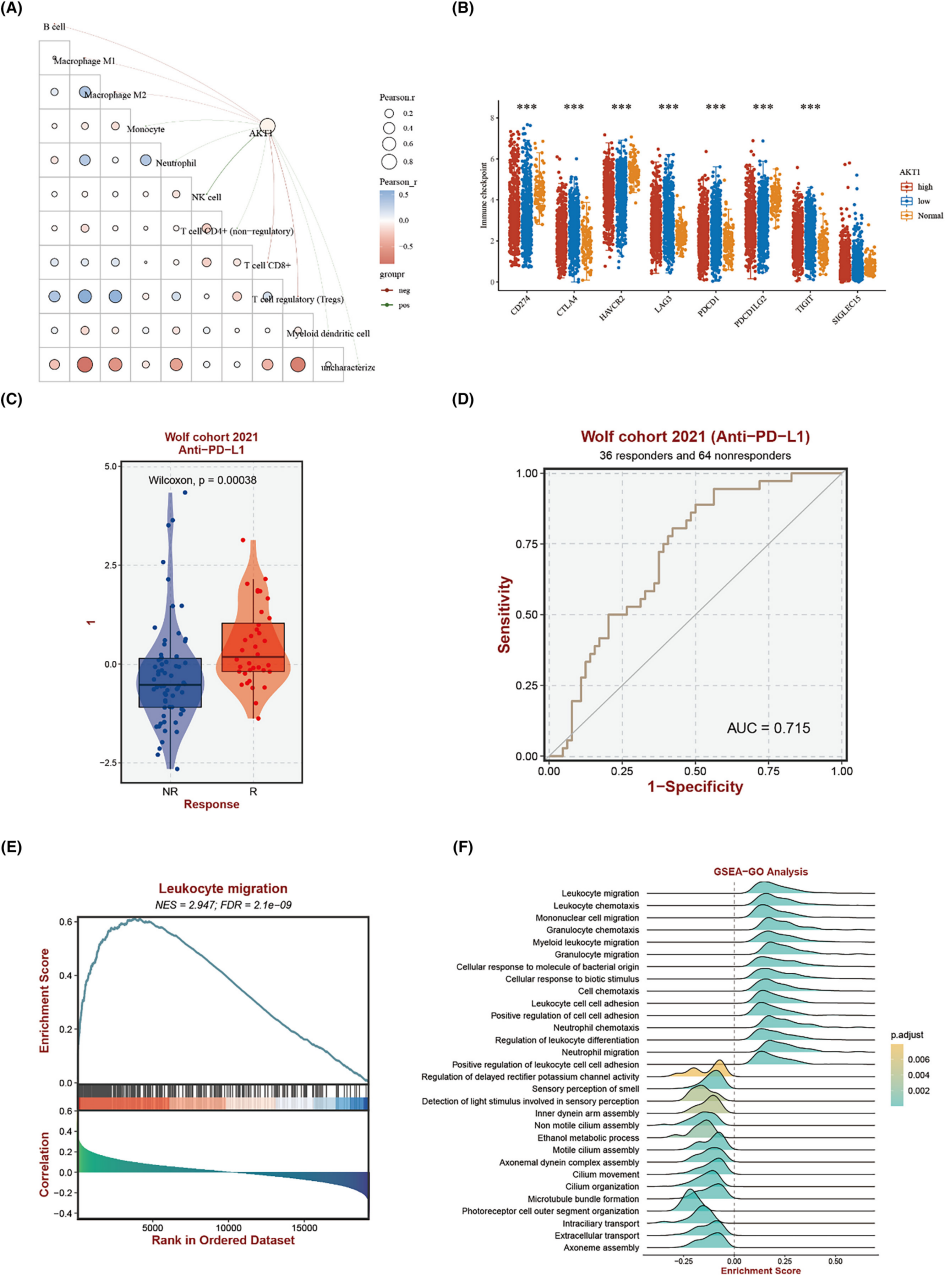
Patterns of immune infiltration. (A) Relationship between AKT1 and immune cell infiltration. (B) Relationship between AKT1 and immune checkpoint expression. (C, D) Expression levels of 10 key genes and the prediction ability of immune response in NSCLC. (E, F) GSEA enrichment shows the key immune pathways. (***p < 0.001).

In the realm of immunotherapy response, extensive studies on the 10 essential genes influenced by EHH have shown a marked presence in immune cells, indicating a link to the proliferation of T cells. This discovery led us to further explore the interplay between these crucial genes and the expression levels of immune checkpoints. Our research identified a notable negative association between the gene AKT1 and the presence of T cells (Figure 8A), which is closely intertwined with the expression of immune checkpoints (Figure 8B). This finding suggests that NSCLC patients with increased AKT1 levels may show a more pronounced response to anti‐PD‐L1 therapy (Figure 8C,D). Additionally, GSEA analysis has highlighted that patients with NSCLC who demonstrate a higher expression of this integral gene set tend to attract a more substantial influx of immune cells (Figure 8E,F).

### 3.7 Outcomes of molecular docking investigations

**FIGURE 9:**
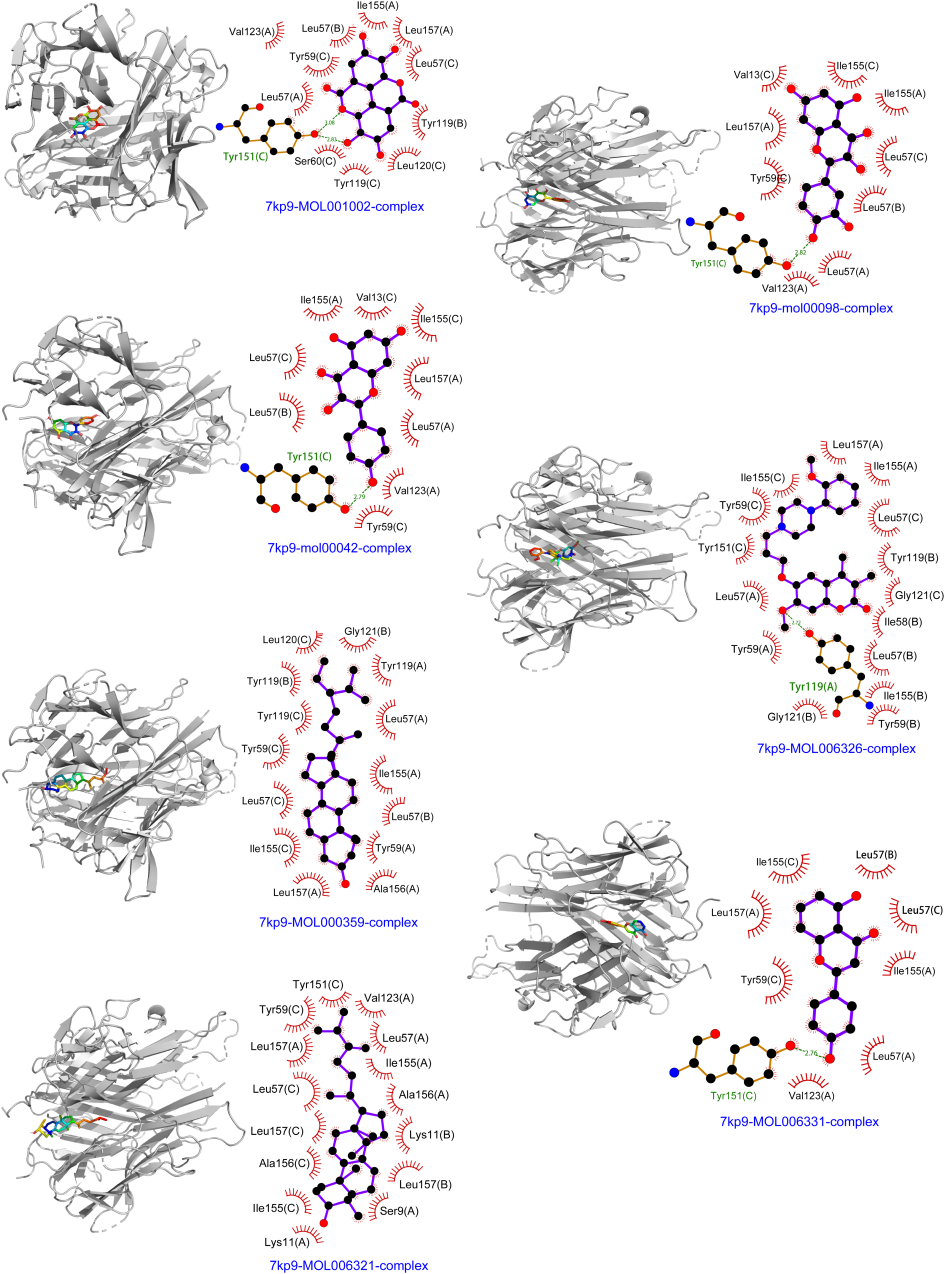
Molecular docking between seven bioactive compounds of EHH and the receptor proteins.

Employing SYBYL‐X 2.1.1 for analysis, it was discerned that a total of seven bioactive compounds seamlessly integrated into the predetermined active site (Figure 9). The molecular docking study's findings explicitly revealed that the ΔG values, indicative of binding free energy in kilocalories per mole, for these seven compounds and the primary target proteins were invariably negative. This emphatically indicates a natural tendency of the ligand molecules to autonomously associate with the receptor proteins. It is worth noting that the calculated values of binding energy in the data were uniformly lower than −5 kJ/mol, thereby reinforcing the evidence of their strong affinity‐based interactions.

### 3.8 EHH induces mitochondrial disruption in NSCLC cells to induce apoptosis

**FIGURE 10:**
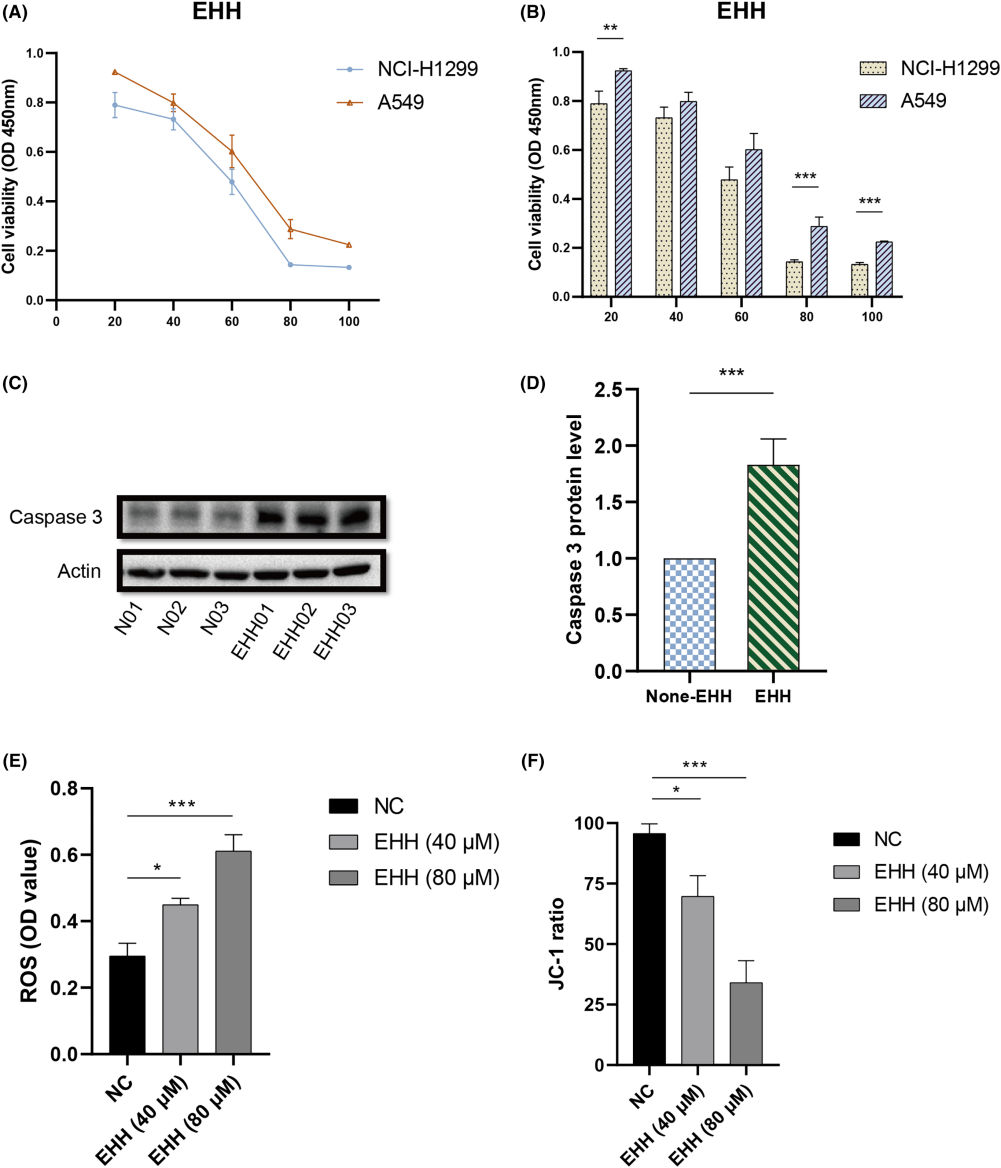
In vitro anti‐NSCLC effects of EHH. (A, B) CCK‐8 assay. (C, D) Western blot detected protein expression after treating with EHH in NCI‐H1299 cell line. (E) ROS level after treating with EHH in NCI‐H1299 cell line. (F) Mitochondrial membrane potential in NCI‐H1299 cells after treating with EHH. (*p < 0.05, **p < 0.01, ***p < 0.001).

**FIGURE 11:**
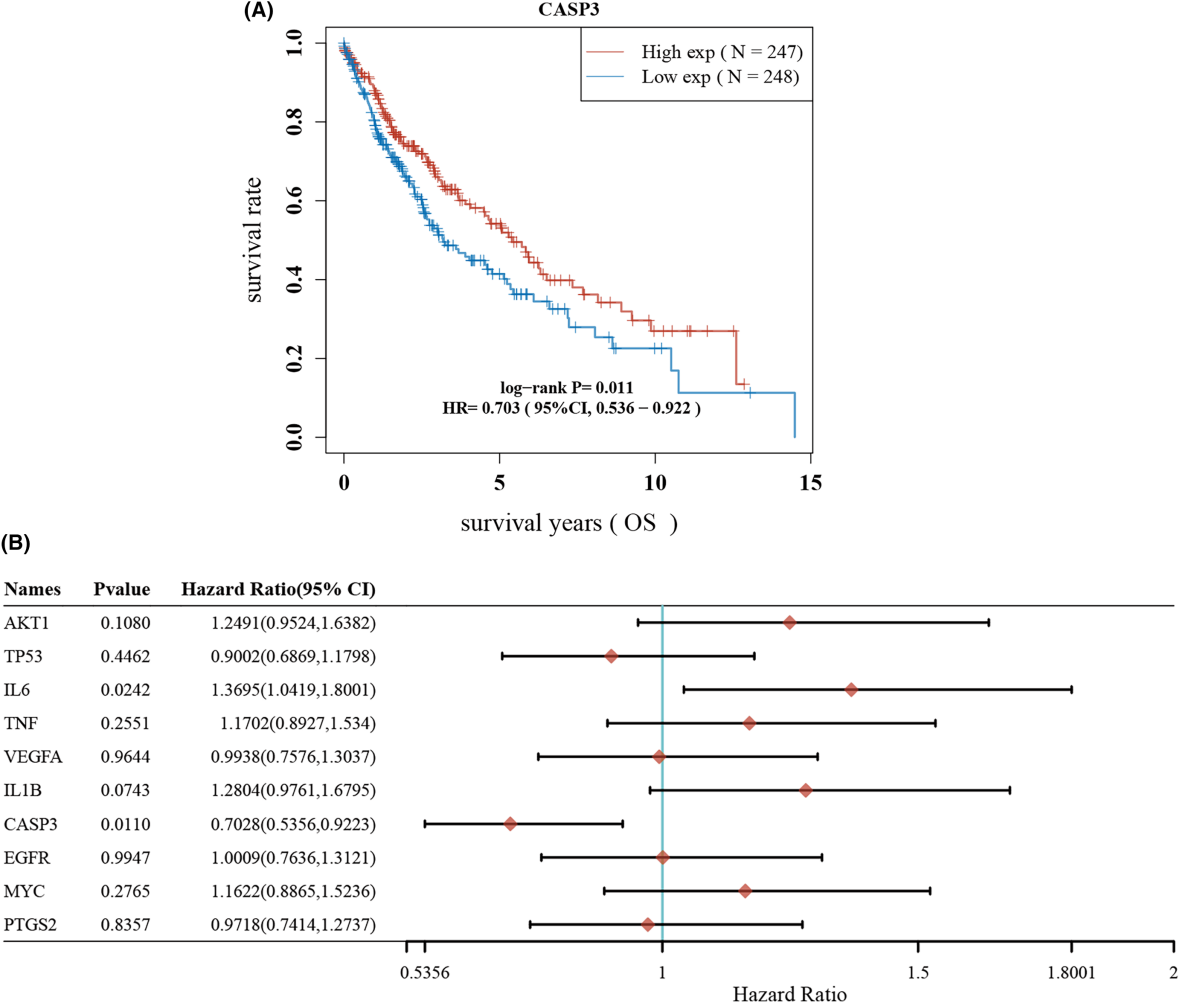
The relationship between CASP 3 and survival in patients with NSCLC. (A) Survival analysis indicates that CASP 3 is indicative of improved survival outcomes in patients with NSCLC. (B) Among the 10 central genes analysed, CASP3 stands out as the sole gene correlated with prognosis.

**FIGURE 12:**
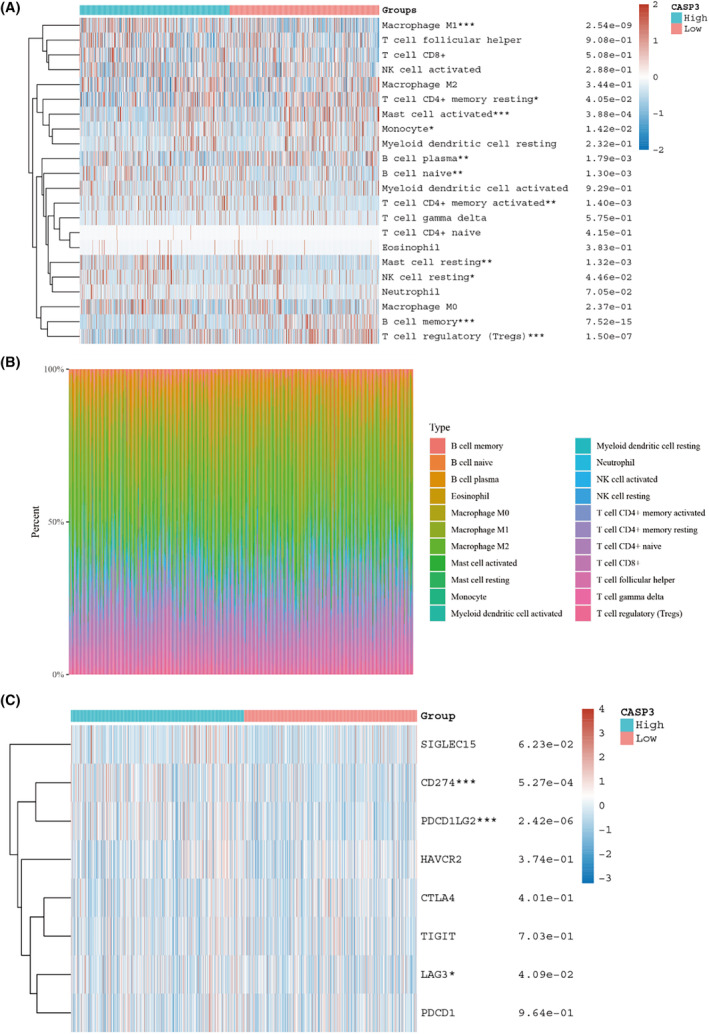
Association between CASP3 and immune cell infiltration levels. (A, B) Immune infiltration landscapes in NSCLC patients exhibiting high versus low CASP3 expression. (C) Correlation of CASP3 expression levels with immune checkpoint expression (*p < 0.05, **p < 0.01, ***p < 0.001).

In this study, our observations identified CASP3 as a crucial node in the interaction network, as illustrated in Figure 3B. Moreover, it was noted that CASP3's expression was diminished in NSCLC tumour cells, as seen in Figure 6. This led us to propose that Caspase 3 may have a significant impact on the cytotoxic effects induced by EHH. To examine this proposition, we treated two NSCLC cell lines with varying doses of EHH over a 48‐h period. The results, obtained through CCK‐8 assays, revealed that EHH's inhibitory impact on both NSCLC cell lines was dose‐dependent, as depicted in Figure 10A. Remarkably, the NCI‐H1299 cells showed a higher level of susceptibility to EHH than A549 cells, as evidenced in Figure 10B. Subsequent investigations into the effects of EHH treatment on NCI‐H1299 cells disclosed variations in Caspase 3 expression (Figure 10C). A marked increase in Caspase 3 protein levels was noted following EHH treatment, significantly surpassing those in the control group (*p* < 0.001; Figure 10D). These findings imply that EHH enhances Caspase 3 protein accumulation, thereby initiating apoptosis in NSCLC cells and potentially hindering the progression of the disease. Moreover, our studies uncovered that EHH markedly augments mitochondrial ROS production in NCI‐H1299 cells, along with inducing changes in mitochondrial membrane potential (Figure 10E,F). This indicates that EHH may act as a potential agent for inducing mitochondrial dysfunction, shedding light on the mechanism behind its role in promoting apoptosis in NSCLC cells. Besides, we observed a strong association between Caspase 3 expression and the prognosis of NSCLC (Figure 11A). High Caspase 3 expression is indicative of a better overall survival in NSCLC patients when compared to low Caspase 3 expression (Figure 11A). The different expression levels of CASP3 divided the categorization of NSCLC patients into two groups. We observed significant differences in Macrophage M1 and T‐cell regulatory (Figure 12A,B). Furthermore, there are variations in the expression levels of immune checkpoint markers such as CD274 and PDCD1LG2 (Figure 12C).

**FIGURE 13:**
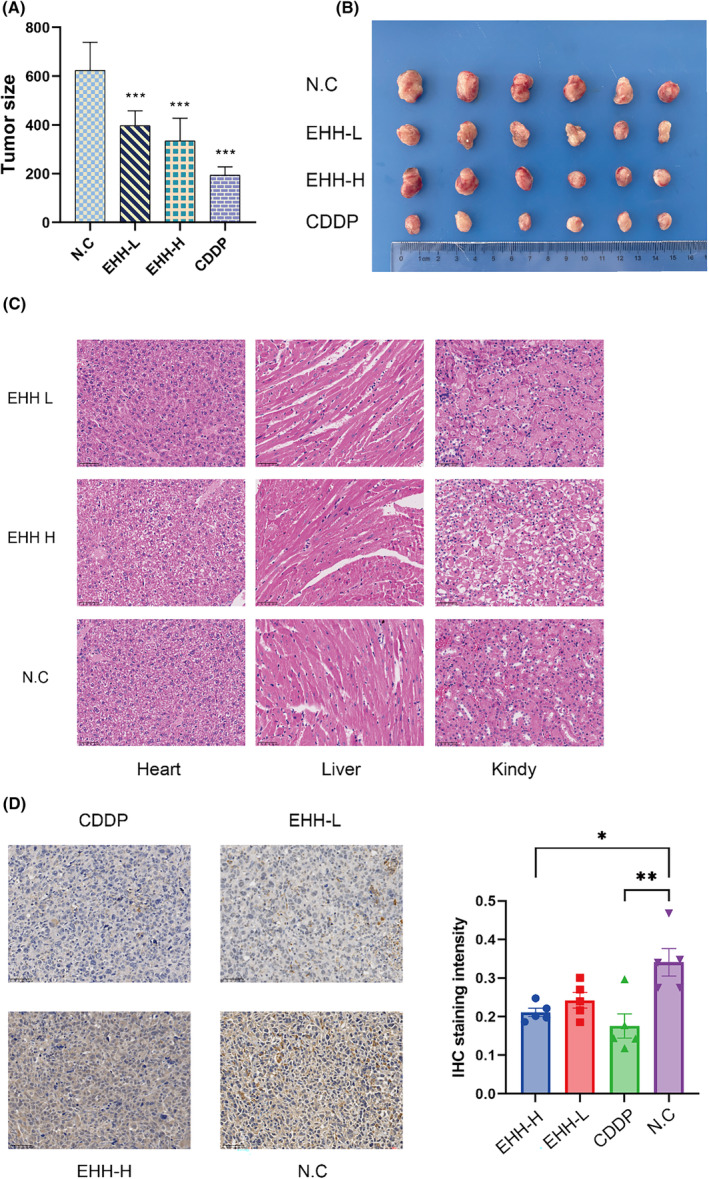
In vivo anti‐NSCLC effects of EHH. (A, B) Subcutaneous tumour formation test. (C) HE staining of heart, kidney and liver. (EHH‐H: high‐dose EHH; EHH‐L: low‐dose EHH; N.C: control group). (D) Immunohistochemistry confirms the anti‐tumour effect of EHH (*p < 0.05, **p < 0.01, ***p < 0.001).

Subsequent to this, in vivo experiments were conducted to assess the anti‐cancer capability of EHH. Measurements of tumour sizes in subcutaneous models were conducted on Days 6, 9, 12 and 15 of the investigation (Figure 13A). A notable reduction in tumour volume was observed in the rodents receiving a higher dosage of EHH compared to those in the control group (Figure 13B). Further analysis, particularly Ki‐67 staining, revealed a decrease in Ki‐67 expression in the EHH‐treated specimens, further confirming EHH's effectiveness in cancer suppression (Figure 13D). Histological analysis using HE staining was carried out to evaluate EHH's potential toxic effects. This analysis indicated significant alleviation of tissue damage in the heart, kidneys and liver following EHH treatment, highlighting EHH's advantageous safety profile in vivo (Figure 13C).

## 4 DISCUSSION

The global prevalence of lung cancer, as the most common malignancy, remains undiminished, coupled with distressingly high mortality rates.^34^ Research in the realm of NSCLC has undergone considerable transformation, underscoring the complex interplay between cancer progression and mitochondrial impairment. Recent investigations have brought to light the significance of mitochondrial irregularities in NSCLC's aetiology, highlighting their contribution to cancer cells' aberrant metabolic and apoptotic activities. This developing viewpoint is in concordance with our observations, which propose a crucial function for Euphorbiae Humifusae Herba (EHH) in influencing mitochondrial pathways, thereby presenting novel therapeutic possibilities. T therapeutic strategies encompass a diverse array of treatments, such as surgical procedures, chemotherapeutic regimens, radiation therapy, targeted molecular therapies and immunotherapeutic approaches. Recent years have witnessed notable achievements in targeted molecular therapy, yet this field faces numerous challenges, including metastatic disease progression, emergence of resistance to therapeutic drugs and various adverse effects related to the treatment. These challenges significantly impact the quality of life of individuals undergoing treatment. The development of anti‐cancer strategies and experimental medical research is progressing rapidly, marking significant milestones in the management of tumours. The field of bioinformatics, particularly, has seen swift progress, offering new avenues for cancer diagnosis, prognosis and therapy. These include innovations like single‐cell sequencing^35^, ^36^, ^37^, ^38^, ^39^, ^40^ and machine learning applications.^41^, ^42^ Such advancements are critical not only in reducing the side effects associated with chemotherapy and radiotherapy but also in enhancing the precision of molecular targeting methods, thereby contributing to improved patient survival outcomes.

The advancement of multi‐omics has significantly enhanced our understanding of diseases. Murtaza and colleagues identified biological mechanisms by constructing PPI networks.^43^ Intriguingly, based on PPI networks, Yang et al.^44^ developed a deep learning model for predicting responses to tumour pharmacotherapy. Our research delves into the multi‐omic integration to identify EHH‐dependent anti‐tumour signalling pathways. The construction of a PPI network using EHH‐targeted proteins unravels the complex interaction within NSCLC's immune microenvironment. Osimertinib, a therapeutic for NSCLC harbouring specific mutations, was examined. It was found that targeting AKT1 could circumvent resistance to osimertinib and induce apoptosis in NSCLC cells.^45^ Cytokines, pivotal in physiological processes, are implicated in tumorigenesis.^46^, ^47^ Beyond chemotherapy, NSCLC stem cells with elevated IL‐6 expression were discovered to resist radiation‐induced apoptosis.^48^ The TP53 gene, frequently altered in NSCLC patients,^49^ enhances tumour cell proliferation and invasiveness when activated; its inhibition markedly induces apoptosis in NSCLC cells.^50^ Consistent with these findings, our study demonstrates that EHH regulates genes such as AKT1, IL6 and TP53 (Figure 4A). Significantly, EHH's interaction with cells notably inhibited A549 cell proliferation (Figure 10B) and induced mitochondrial ROS accumulation and membrane potential dysregulation (Figure 10E,F), culminating in apoptosis of A549 cells (Figure 10C). Hence, this suggests that EHH induces mitochondrial damage and promotes apoptosis in NSCLC cells by targeting key genes like AKT1, IL6 and TP53.

The application of single‐cell sequencing in cancer research has revolutionized our comprehension of the biological characteristics and dynamics of cancerous lesions.^51^, ^52^ Through single‐cell analysis and annotation of NSCLC, we observed a notable pattern: CASP3 expression was markedly elevated in CD8 T cells but comparatively lower in NSCLC cells (Figure 6). Interestingly, Paula et al. noted that mice with diminished CASP3 expression exhibited reduced anti‐tumour immunity,^53^ elucidating, from another perspective, why patients with high‐risk NSCLC demonstrate enhanced immune responses (Figure 8C). Furthermore, our investigations revealed that NSCLC patients with elevated CASP3 levels showed increased expression of PD‐L2 (Figure 12C), a protein predominantly expressed by certain immune cells like macrophages and dendritic cells. It inhibits T‐cell activity by binding to PD‐1.^54^ Inhibitors targeting the PD‐1/PD‐L1 and PD‐L2 pathways have emerged as effective treatments for specific cancer types,^55^ suggesting that combining EHH with immunotherapy could potentiate anti‐tumour efficacy. Additionally, the single‐cell analysis underscores the differential expression of these genes across various cell types, particularly immune cells, emphasizing EHH's role in altering the tumour microenvironment.

In the evolving landscape of lung cancer therapy, resistance to medicines presents an inescapable challenge. Our research adopts a network pharmacology approach to shed light on the complex interplay of active ingredients, molecular targets and pathways, thereby identifying the molecular actors and targets through which EHH exerts its influence in treating NSCLC. An in‐depth examination of 13 bioactive compounds in EHH reveals a complex array of NSCLC‐related targets, notably including fundamental components such as quercetin, kaempferol, ellagic acid and sitosterol. Quercetin is recognized for its anti‐inflammatory, antioxidative and anti‐cancer properties.^56^ Studies by Ward and colleagues on prostate cancer cells carrying p53 mutations demonstrated that quercetin significantly suppresses the PI3K/Akt pathway and stimulates the accumulation of ROS, thus enhancing the expression of apoptosis‐related proteins.^57^ Kaempferol, a molecule with potential anti‐tumour activity, has been shown to modulate the expression of Ki67 in tumour tissues,^58^ suggesting that the observed alterations in Ki67 expression following EHH treatment in NSCLC could be attributed to kaempferol (Figure 13D). The combination of ellagic acid with chemotherapy has been found to exert a synergistic anti‐tumour effect,^59^ leading to future considerations in our research to compare cisplatin with EHH directly (Figure 11A). Intriguingly, our findings indicate that the target genes of EHH are related to resistance against cisplatin (Figure 7A), prompting further investigations into the synergistic effects between EHH and cisplatin. In sum, these molecules play a pivotal role in the EHH composition, signifying their crucial function in facilitating an effective anti‐NSCLC action.

While the extensive utilization of EHH has been well‐documented, the precise mechanisms underlying its anti‐cancer properties remain unclear. Utilizing network pharmacology and bioinformatics methodologies, this study reveals a group of 133 genes distinctly associated with EHH's active elements, all linked to NSCLC. Through comprehensive GO functional analysis of overlapping genes, a suite of 225 enriched processes was identified. Studies have indicated that EHH can inhibit NF‐κB activity and reduce the expression of TNFα‐induced matrix metalloproteinase (MMP)‐9 mRNA, thereby attenuating the migratory capabilities of breast cancer cells.^6^ In our investigation, EHH predominantly modulated TNF and MMP9, with secondary modulation of NFKBIA (Figure 4A). This suggests that EHH's opposition to A549 cells is partly achieved through the TNF/MMP9 pathway. These genes form a considerable segment of the targets related to the relevant active elements of EHH. This finding highlights the deep connections between the active constituents of EHH and NSCLC.

Subsequent analysis using the Kyoto Encyclopedia of Genes and Genomes (KEGG) has elucidated a spectrum of processes modulated by EHH in NSCLC, including oncogenesis, lipid‐related metabolic activities, atherosclerosis development, as well as sequences linked to prostate cancer, the genesis of chemical carcinogenesis and the initiation of receptor activities (Figure 7A). This complex network is further augmented by routes that demonstrate the impact of proteoglycans in cancer progression, signalling mechanisms through the HIF‐1 axis, defiance against platinum‐based therapeutic agents, countermeasures to inhibitors of epidermal growth factor receptor tyrosine kinase and the nuances of relaxin signalling pathways. Predominantly, these routes integrate with domains encompassing tumour genesis, regulation of cell cycles and the coordination of immune responses (Figure 7B).

In brief, our study illuminates the importance of integrating single‐cell and multi‐omic analyses to identify novel anti‐tumour mechanisms, particularly in the context of NSCLC. The identified EHH‐dependent pathways and biomarkers offer promising avenues for developing targeted immunotherapy against mitochondrial dysfunction in NSCLC. However, certain limitations are inherent within the confines of this research. For instance, the evaluation of the immune microenvironment and immune infiltration was conducted utilizing transcriptomic data as opposed to flow cytometry techniques, potentially leading to discrepancies between our findings and the actual levels of immune infiltration. Moreover, our experimental outcomes are confined to cellular and preclinical stages, which might not accurately depict the therapeutic efficacy of EHH in clinical NSCLC patients. Future research should focus on validating these findings in clinical settings and exploring the potential of EHH as a therapeutic agent in other cancer types.

## 5 CONCLUSION

This study lays a foundation for a new era in mitochondrial‐targeted cancer therapy, potentially revolutionizing EHH as a new NSCLC treatment strategy.

## AUTHOR CONTRIBUTIONS

**Chengcheng Zhang:** Data curation (equal); formal analysis (equal); methodology (equal); software (equal); validation (equal); visualization (lead); writing – original draft (equal). **Xiaoxue Zhao:** Formal analysis (equal); methodology (equal); visualization (equal); writing – original draft (equal). **Feng Li:** Formal analysis (equal); software (equal); visualization (equal); writing – original draft (equal). **Jingru Qin:** Data curation (equal); investigation (equal); resources (equal). **Lu Yang:** Data curation (equal); methodology (equal); resources (equal). **Qianqian Yin:** Formal analysis (equal); visualization (equal). **Yiyi Liu:** Formal analysis (equal); software (equal); visualization (equal). **Zhiyao Zhu:** Formal analysis (equal); software (equal); visualization (equal). **Fei Zhang:** Data curation (equal); investigation (equal); validation (equal). **Zhongqi Wang:** Conceptualization (equal); project administration (equal); resources (equal); supervision (equal); validation (equal); writing – review and editing (equal). **Haibin Liang:** Conceptualization (equal); funding acquisition (equal); investigation (equal); project administration (equal); resources (equal); supervision (equal); validation (equal); writing – review and editing (equal).

## FUNDING INFORMATION

This study was supported in part by Shanghai Municipal Health Commission, Health Youth Talent Project (2022YQ028), Shanghai Hospital Development Center (SHDC2020CR4050), Shanghai Shenkang Hospital Development Center (SHDC12020123), Shanghai Medical Innovation Research Project of ‘Science and Technology Innovation Action Plan’ (No.20Y21902300) and LongHua Hospital Medical Scholars Scheme (YM2021023).

## CONFLICT OF INTEREST STATEMENT

The authors declare that the research was conducted in the absence of any commercial or financial relationships that could be construed as a potential conflict of interest.

## PATIENT CONSENT FOR PUBLICATION

The patient cohort used in this study is derived from a public dataset (TCGA‐NSCLC); therefore, patient consent for publication is not required.

## Supporting information


Figure S1.

Table S1.

Table S2.

## Data Availability

The datasets used in this study can be accessed through the TISCH2 (http://tisch.comp‐genomics.org/) portal. All raw data are available at: https://www.jianguoyun.com/p/DQbtuAsQovD_ChiIwZUFIAA.
