# Correction to ‘Integrating Single‐Cell and Multi‐Omic Approaches Reveals Euphorbiae Humifusae Herba‐Dependent Mitochondrial Dysfunction in Non‐Small‐Cell Lung Cancer’

**DOI:** 10.1111/jcmm.70305

**Published:** 2024-12-27

Zhang C, Zhao X, Li F, Qin J, Yang L, Yin Q, et al. (2024), Integrating Single‐Cell and Multi‐Omic Approaches Reveals Euphorbiae Humifusae Herba‐Dependent Mitochondrial Dysfunction in Non‐Small‐Cell Lung Cancer. *Journal of Cellular and Molecular Medicine* 2024; 28:e18317. doi:10.1111/jcmm.18317

The Institutional Review Board Statement was inadvertently removed from the manuscript during article processing.

This should read as follows:

The animal study protocol was approved by the Ethics Committee of Experimental Animal Ethics

509 Committee of Shanghai University of Traditional Chinese Medicine for studies involving animals

510 (NO. PZSHUTCM221010008).

We apologise for this error.

