# Supplementary figures and images for: Integrating single‐cell and multi‐omic approaches reveals Euphorbiae Humifusae Herba‐dependent mitochondrial dysfunction in non‐small‐cell lung cancer
Source: J Cell Mol Med. 2024 May 27;28(10):e18317. doi: 10.1111/jcmm.18317 (PMC11129731; doi:10.1111/jcmm.18317)

**
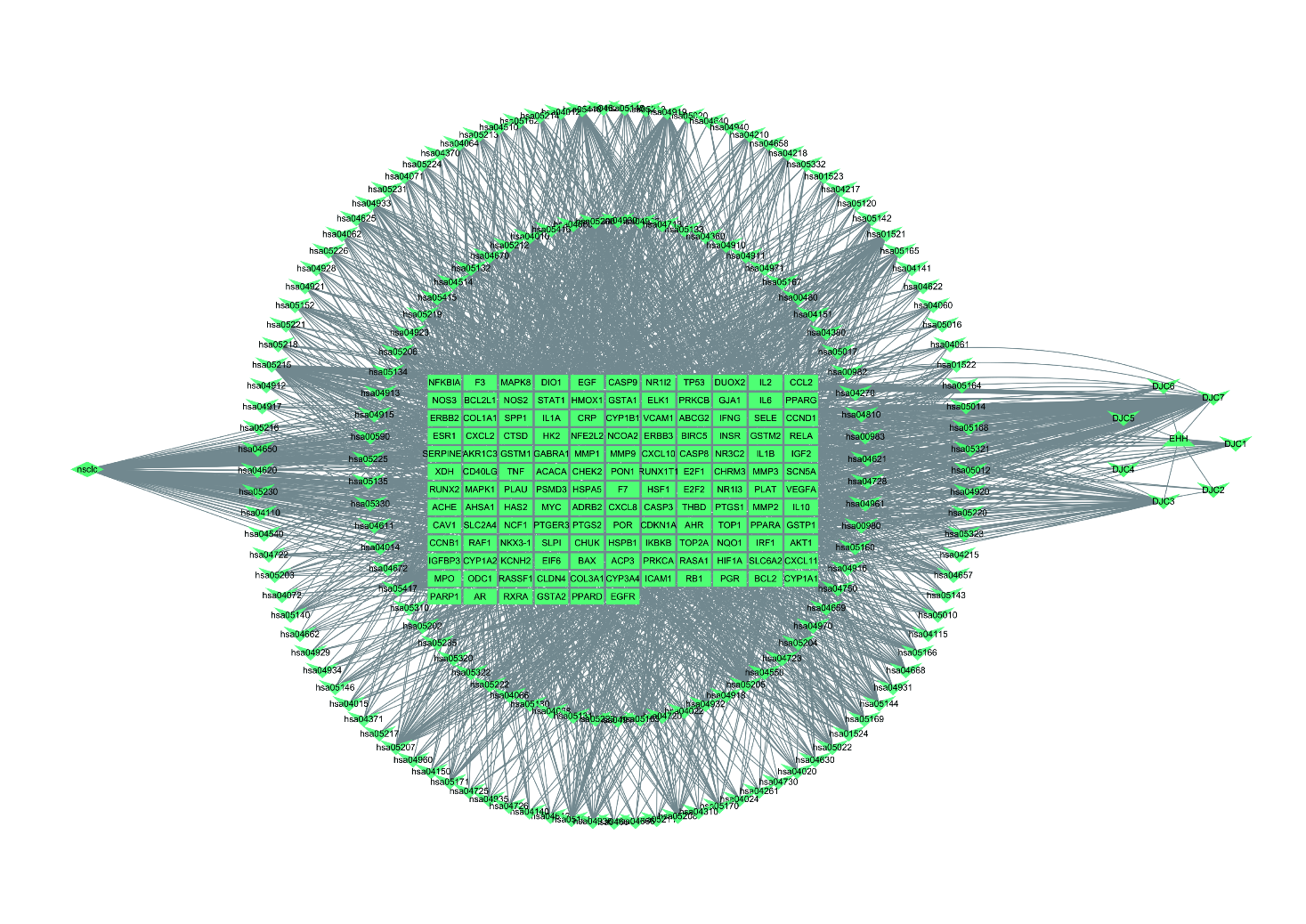
**

**Figure S1:** EHH regulatory network.

Supplement: Supplementary file 1 — Figure S1. [file JCMM-28-e18317-s003.docx]
